# SPINEPS—automatic whole spine segmentation of T2-weighted MR images using a two-phase approach to multi-class semantic and instance segmentation

**DOI:** 10.1007/s00330-024-11155-y

**Published:** 2024-10-29

**Authors:** Hendrik Möller, Robert Graf, Joachim Schmitt, Benjamin Keinert, Hanna Schön, Matan Atad, Anjany Sekuboyina, Felix Streckenbach, Florian Kofler, Thomas Kroencke, Stefanie Bette, Stefan N. Willich, Thomas Keil, Thoralf Niendorf, Tobias Pischon, Beate Endemann, Bjoern Menze, Daniel Rueckert, Jan S. Kirschke

**Affiliations:** 1 Department of Diagnostic and Interventional Neuroradiology, School of Medicine and Health, Technical University of Munich, Munich, Germany; 2 Institut Für KI Und Informatik in Der Medizin, Klinikum Rechts Der Isar, Technical University of Munich, Munich, Germany; 3 Department of Diagnostic and Interventional Radiology, Pediatric Radiology and Neuroradiology, University Medical Center Rostock, Rostock, Germany; 4 Department of Quantitative Biomedicine, University of Zurich, Zurich, Switzerland; 5 Helmholtz AI, Helmholtz Munich, Neuherberg, Germany; 6 TranslaTUM—Central Institute for Translational Cancer Research, Technical University of Munich, Munich, Germany; 7 Department of Diagnostic and Interventional Radiology, University Hospital Augsburg, Augsburg, Germany; 8 Centre for Advanced Analytics and Predictive Sciences, Augsburg University, Augsburg, Germany; 9 Institute of Social Medicine, Epidemiology and Health Economics, Charité—Universitätsmedizin Berlin, Berlin, Germany; 10 Institute of Clinical Epidemiology and Biometry, University of Würzburg, Würzburg, Germany; 11 State Institute of Health I, Bavarian Health and Food Safety Auhtority, Erlangen, Germany; 12 Max Delbrück Center for Molecular Medicine in the Helmholtz Association, Berlin, Germany; 13 Molecular Epidemiology Research Group, Max-Delbrueck-Center for Molecular Medicine in the Helmholtz Association (MDC), Berlin, Germany; 14 Biobank Technology Platform, Max-Delbrueck-Center for Molecular Medicine in the Helmholtz Association (MDC), Berlin, Germany; 15 Charité—Universitätsmedizin Berlin, Corporate member of Freie Universität Berlin and Humboldt-Universität zu Berlin, Berlin, Germany; 16 Department of Computing, Imperial College London, London, UK

**Keywords:** Spine, Magnetic resonance imaging, Intervertebral disc, Vertebral body, Deep learning

## Abstract

**Objectives:**

Introducing SPINEPS, a deep learning method for semantic and instance segmentation of 14 spinal structures (ten vertebra substructures, intervertebral discs, spinal cord, spinal canal, and sacrum) in whole-body sagittal T2-weighted turbo spin echo images.

**Material and methods:**

This local ethics committee-approved study utilized a public dataset (train/test 179/39 subjects, 137 female), a German National Cohort (NAKO) subset (train/test 1412/65 subjects, mean age 53, 694 female), and an in-house dataset (test 10 subjects, mean age 70, 5 female). SPINEPS is a semantic segmentation model, followed by a sliding window approach utilizing a second model to create instance masks from the semantic ones. Segmentation evaluation metrics included the Dice score and average symmetrical surface distance (ASSD). Statistical significance was assessed using the Wilcoxon signed-rank test.

**Results:**

On the public dataset, SPINEPS outperformed a nnUNet baseline on every structure and metric (e.g., an average over vertebra instances: dice 0.933 vs 0.911, *p* < 0.001, ASSD 0.21 vs 0.435, *p* < 0.001). SPINEPS trained on automated annotations of the NAKO achieves an average global Dice score of 0.918 on the combined NAKO and in-house test split. Adding the training data from the public dataset outperforms this (average instance-wise Dice score over the vertebra substructures 0.803 vs 0.778, average global Dice score 0.931 vs 0.918).

**Conclusion:**

SPINEPS offers segmentation of 14 spinal structures in T2w sagittal images. It provides a semantic mask and an instance mask separating the vertebrae and intervertebral discs. This is the first publicly available algorithm to enable this segmentation.

**Key Points:**

***Question*** *No publicly available automatic approach can yield semantic and instance segmentation masks for the whole spine (including posterior elements) in T2-weighted sagittal TSE images*.

***Findings*** *Segmenting semantically first and then instance-wise outperforms a baseline trained directly on instance segmentation. The developed model produces high-resolution MRI segmentations for the whole spine*.

***Clinical relevance*** *This study introduces an automatic approach to whole spine segmentation, including posterior elements, in arbitrary fields of view T2w sagittal MR images, enabling easy biomarker extraction, automatic localization of pathologies and degenerative diseases, and quantifying analyses as downstream research*.

## Introduction

MRI is commonly used to evaluate the spine in clinical practice, providing diagnostically valuable data on intervertebral disc (IVD) degeneration, vertebrae pathologies, and spinal canal/cord structures [1].

Segmentation is well-established in imaging techniques such as computed tomography (CT). For instance, it can be utilized in surgical and radiotherapy planning [2]. In spinal MRI, segmentation is less common, primarily due to missing resources as well as complex anatomy and poor visualization of the posterior elements.

However, whole spine segmentation in MRI enables the automatic extraction of biomarkers, [3] the automatic detection of degenerative diseases such as IVD degeneration with Pfirrmann gradings, [4, 5] or quantifying tumor load [6]. Additionally, quantitative analysis is enabled, such as determining the level of scoliosis [7]. Thus, if whole spine segmentation is available in one of the most frequently used MRI sequences in clinical routine, such as T2w sagittal turbo spin echo (TSE), it can improve the quality of radiologic assessments and reduce workload.

Machine learning is an established tool for solving the problem of semantic or instance segmentation [8]. However, there is currently no automatic approach for MRI images that segments the whole spine, including posterior elements like the spinous processes of vertebrae. Most existing methods for T2w image segmentation are limited to the lumbar region and, therefore, are not designed to segment the whole spine [9–14]. This can mainly be attributed to the fact that MRIs have a low-resolution plane compared to CTs, and structures such as the posterior elements are more difficult to distinguish. This imposes a significant workload on radiologists who perform manual annotations on MRI. Such annotations are usually required to train automatic segmentation approaches.

In contrast, segmentation is well-established for CT imaging [15–17]. To overcome the issue of manual annotations, Graf et al [18] successfully used image translation to create artificial CT images from MR images. They used existing segmentation models for CT to create segmentation masks for MRIs and showed that this translation works well enough to transfer CT-level segmentation masks into MRIs. Our approach combines these CT-level annotations with existing MRI-specific ones to train our models without manually annotating a single MRI. For this, the U-Net architecture, [19] the most common deep learning approach for segmentation tasks, is used. It is a convolutional neural network designed for image segmentation, characterized by its encoder-decoder structure and skip connections. However, we observed from existing models that they struggle with instance segmentation, as the different instance labels belong to the same semantic structure and thus look similar to the model, exemplarily described by Isensee et al [20]. This is unacceptable if such segmentation masks are used for statistical analysis, registration, or medical intervention. This study addresses this issue by first segmenting semantically, allowing us to use spatially relative instance labels for training and inference. Moreover, the availability of both semantic and instance masks enables downstream tasks interested in the semantic structure and the localization of individual instances, such as fracture detection [21].

The purpose of this study is to present a spinal phase-wise imaging network for paired segmentation (SPINEPS), a two-phase approach to segment 14 spinal structures in the cervical, thoracic, and lumbar regions of T2w sagittal images, both semantically and instance-wise, to demonstrate how a combination of annotations derived from automated segmentation models and an MR-to-CT image translation technique can be utilized for training, and to make a pre-trained model publicly available to enable researchers to generate spine segmentation masks for their MR datasets.

## Materials and methods

This study utilized three datasets: (1) public data, (2) a prospectively collected external dataset (participants gave informed consent), and (3) a retrospectively collected in-house dataset (waived informed consent by the local ethics committee, 593/21 S-NP).

**Fig. 1:**
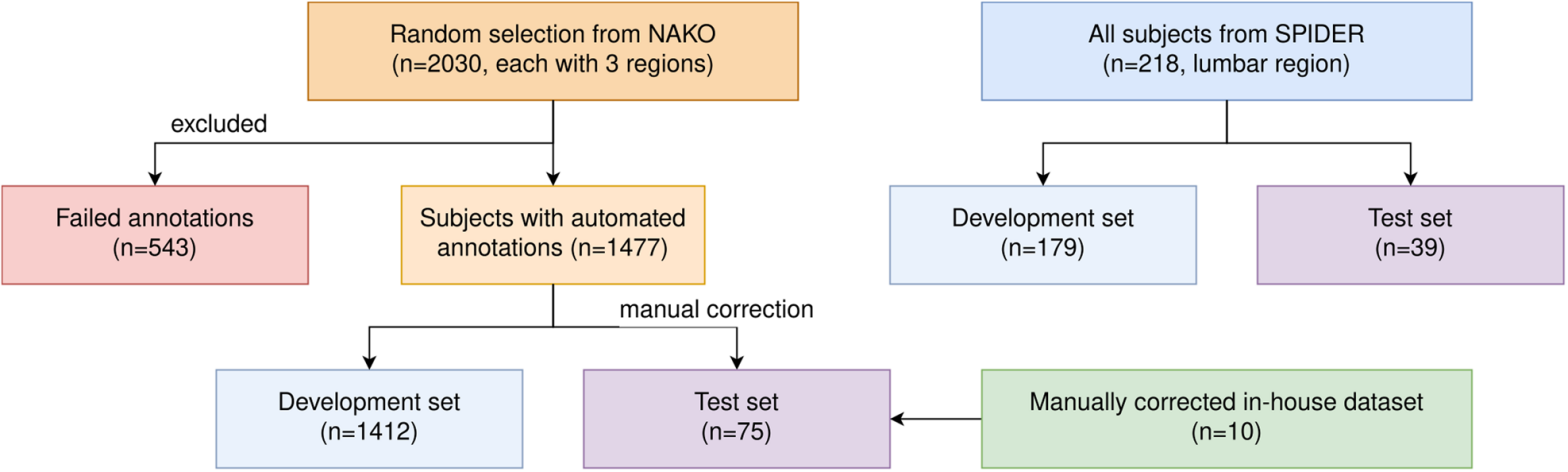
Flow diagram of the investigated study cohort. Flow diagram for subject exclusion from top to bottom for the different datasets (*n* denotes the number of subjects). Only subjects from the NAKO were excluded for which the automated annotation generation approaches failed

In detail, this study utilized the public SPIDER dataset, [22] a random German National Cohort (NAKO) subset, [23] and an in-house dataset (Table 1). A random test split of 18% (39/218) subjects from SPIDER was used. The ratio of train/test for SPIDER is derived from a previous study [22]. For a second test split, 65 random subjects from the NAKO and the images of the in-house dataset of ten subjects underwent manual correction divided among three experts (J.S., H.S., and B.K.) with 3 years, 3 years, and two years of experience, supervised by an expert (J.S.K.) with 22 years of experience (Fig. 1). The high manual effort and time consumption of this correction process limited the size of this test split. The tool for annotation was ITK-SNAP, [24] a cost-free and simple software for viewing and editing 3D images and segmentations. For training on the NAKO data, no manual annotations were directly utilized. Instead, different automated segmentations were combined as training references.

**Table 1:** Study cohort demographics

	German national cohort [23]	SPIDER dataset [22]	In-house dataset
Subjects, (*n*)	2030	218	10
Modality	T2w sagittal scans	T1w and T2w sagittal scans	T2w sagittal scans
MRI specification	3.0-T TSE	1.5-T and 3.0-T TSE	3.0-T TSE
Date range	2014–2016	2019–2022	2021–2022
Region	Cervical, thoracic, and lumbar	Lumbar only	Cervical, thoracic, and lumbar
Sex, (% female)	49 (990/2030)	63 (137/218)	50 (5/10)
Mean age, (years) ± SD	52 ± 11	N/A	70 ± 19
Age range, (years)	21–72	N/A	20–88
Height range, (m)	1.47–2.02	N/A	N/A
Weight range, (kg)	46–145	N/A	50–110
Subject population	Mostly healthy individuals	Clinical history of (chronic) back pain	Clinical history of fractures or spinal degeneration

Demographics of the utilized cohorts

*SD* standard deviation

### Automated annotations

From a previous study by Streckenbach et al [25] manual annotations containing the vertebra corpus, IVD, spinal canal, and sacrum body semantic masks in 180 NAKO subjects were obtained. We trained a default nnUNet model, a widely recognized and powerful tool for image segmentation, using the suggested hyperparameters [26] to replicate this segmentation. This model, alongside the Spinal Cord Toolbox [27] for spinal cord segmentation, was employed to segment the 2030 NAKO subjects. The Spinal Cord Toolbox encountered software issues we could not fix for 543 subjects, which were excluded from further usage, leaving 1477 subjects (1412 of which were used for training).

Adopting Graf et al’s approach, [18] the NAKO training data of T2w sagittal images were translated into artificial CTs. The Bonescreen SpineR tool (Bonescreen GmbH) based on Sekuboyina et al [28] was used to segment the artificial CTs from the second cervical vertebra (C2) to the last lumbar vertebra. These translated annotations yielded nine vertebrae substructures segmentations (corpus, arcus vertebrae, spinous processus, and processus articulares inferiores, superiores, and costales/transversus, the latter three divided into left and right).

**Fig. 2:**
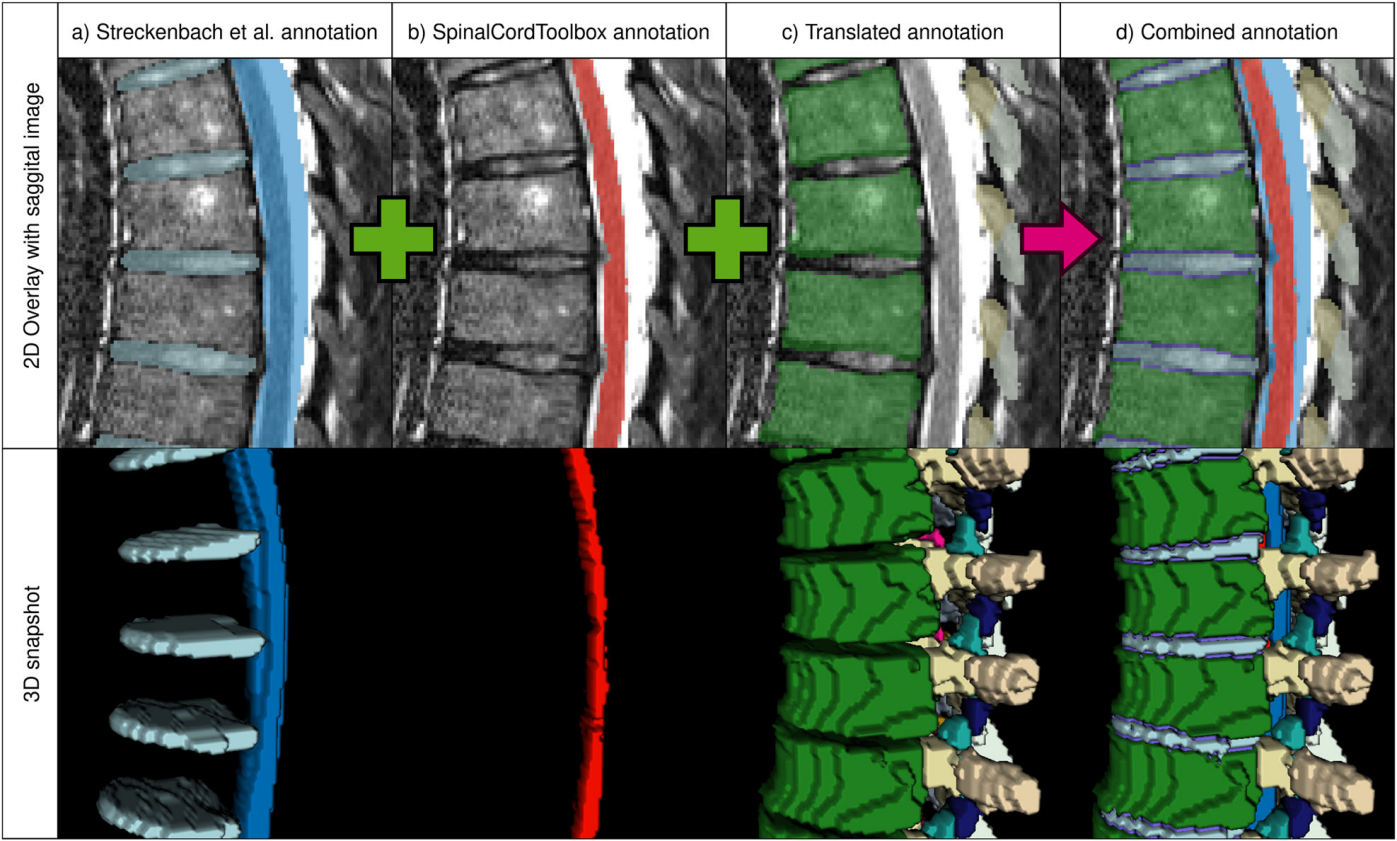
Combination of the automated annotations. Showcase of the three automated annotations and their resulting combined annotation, as a 2D segmentation overlay and a 3D snapshot. **a** Shows the segmentation made with the training data from Streckenbach et al [25], (**b**) the SpinalCordToolbox [27] annotation, (**c**) the annotations derived from translation, and (**d**) the combination of all three. We observed that the manual segmentation from Streckenbach et al [25] is primarily block-shaped and incomplete, while the translated annotations often segmented too many voxels around the vertebra corpus

The different segmentation masks were merged step by step. Translation-based segmentations were added to the Streckenbach-based ones. When adding, voxels already segmented as a different structure were excluded. Next, the spinal cord voxels were incorporated. Finally, holes between the corpus and IVD regions were filled, and the transition pixels were relabeled as endplates (Fig. 2). As the first cervical vertebra (C1) is not segmented in any of our reference masks, our approach cannot segment this particular vertebra.

This resulted in 14 spinal structures: ten for the vertebral substructures (including endplate), spinal canal, spinal cord, sacrum, and IVD. These automated segmentations served as reference annotations for our training with the NAKO train data.

### Segmentation approach

**Fig. 3:**
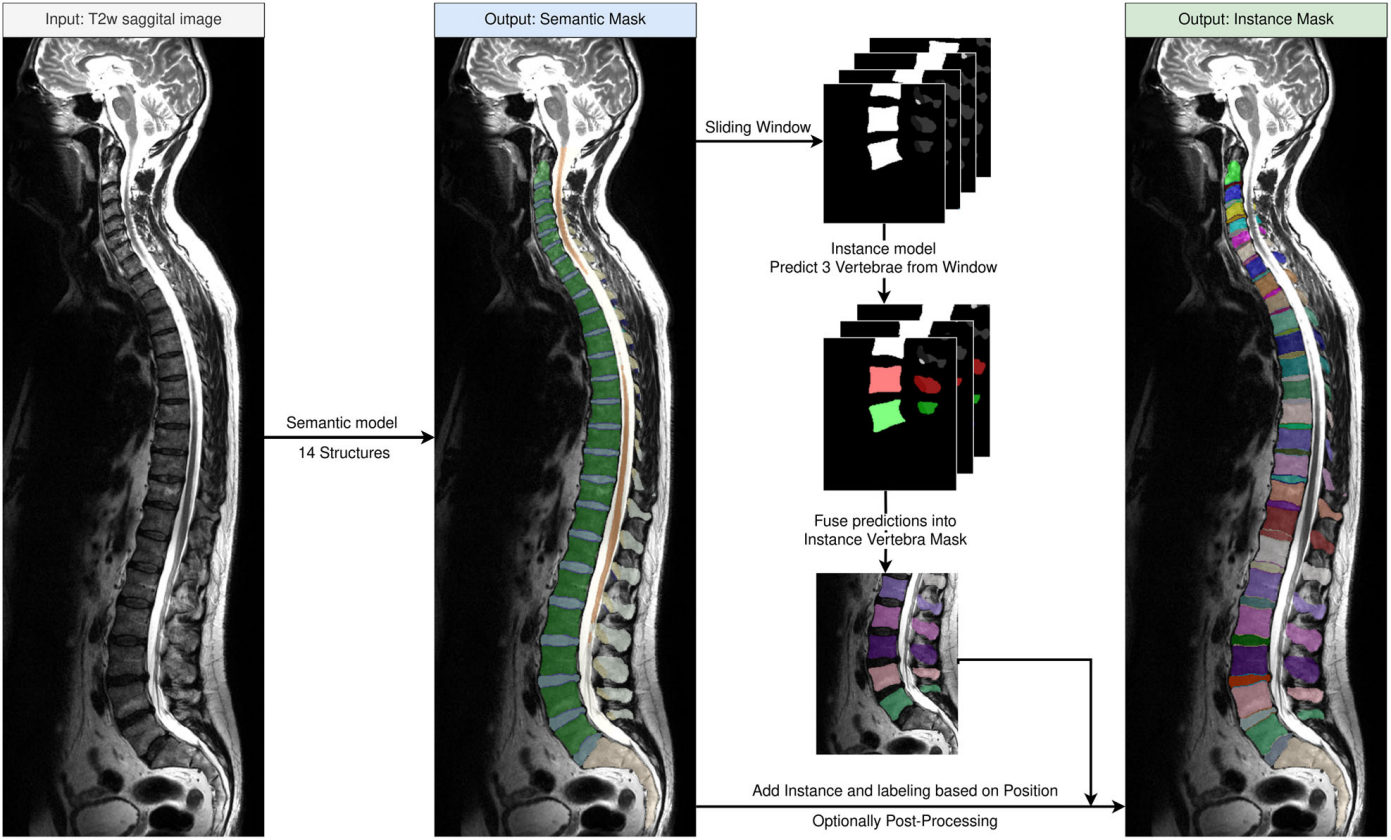
Structure of our segmentation approach. The data flow of our proposed method of inference on new T2w sagittal scans. The semantic model segments 14 different spine structures, regardless of field of view. Then, cutouts are made from the segmentation and fed into the instance model. The results are predictions for the individual vertebrae, which are fused together for the vertebra instance mask. Then, using the first segmentation, each voxel in the instance vertebra mask that is not present in the semantic mask is removed. Finally, IVDs and endplates are matched based on a center of mass analysis. The examples shown are predictions of our model on the whole spine

Our approach operates in two phases (Fig. 3). Initially, a semantic model segments the scan patch-wise into the 14 semantic labels. For this purpose, a nnUNet 3D architecture [26] is employed.

**Fig. 4:**
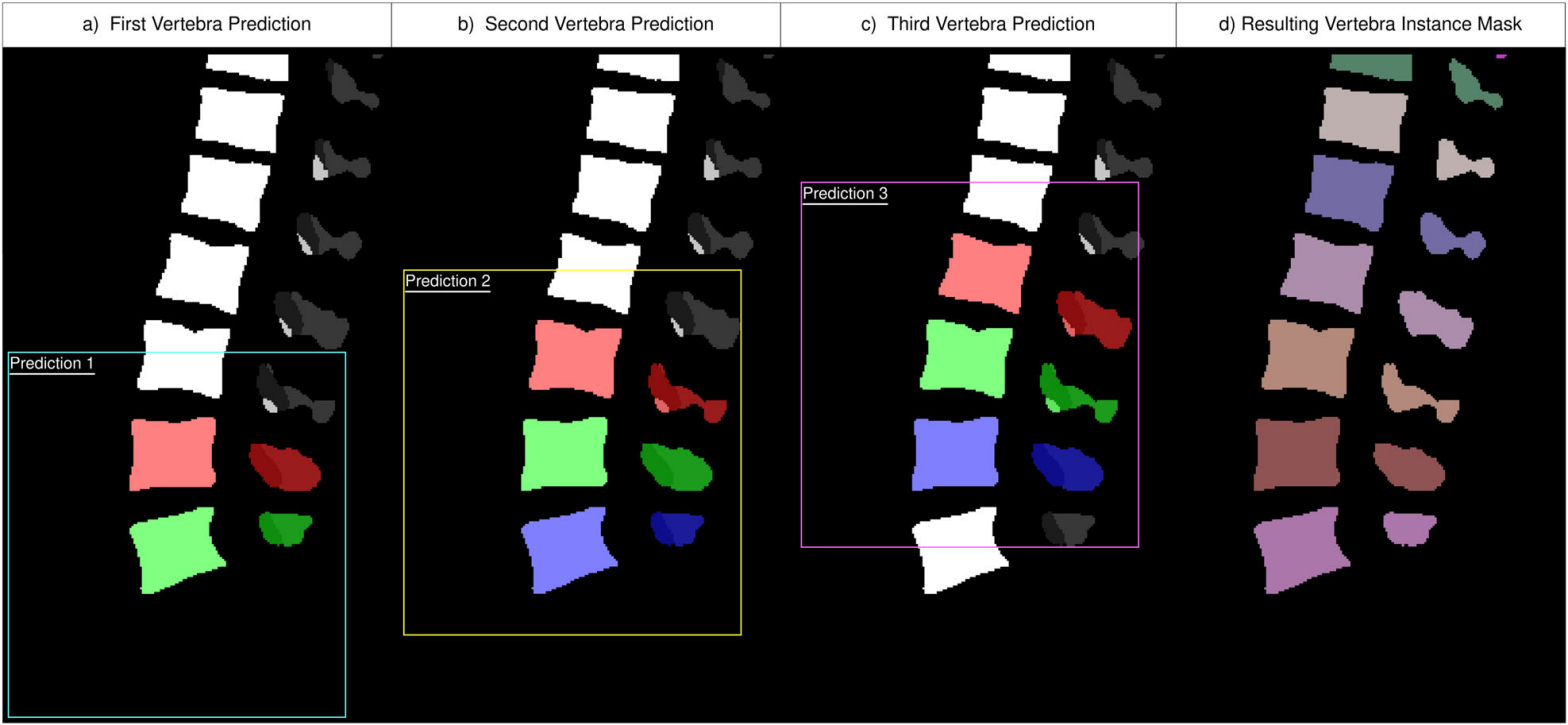
Example of the instance model. Given the semantic segmentation, cutouts of the exact same size are created. Each of those cutouts (colored boxes) is fed into the instance model. **a–c** Show the first three predictions of a semantic input. The instance model always predicts the center vertebra of the cutout (green), as well as the one above (red) and below (blue), if visible. Therefore, assuming no erroneous predictions, we get three predictions for all inner vertebrae and two for the outer ones. For example, the second to last vertebra in the figure is predicted thrice, once in each of the three predictions (red, green, blue, from left to right). The combination of all cutout predictions is combined into a vertebra instance mask (**d**), uniquely labeling each vertebra instance (different colors)

The different instances cannot be trivially computed from this semantic mask, e.g., due to the fusion of vertebrae bodies. Therefore, this study utilizes a sliding window patching approach with a second model (a 3D U-Net [19]) trained to distinguish semantic labels into vertebra instances. This allows us to train the instance segmentation on spatially relative instance labels (i.e., the vertebra instance in the center of the patch) instead of global ones (i.e., the vertebra instance third from the top of the scan). To achieve this, the center of mass position for each vertebra corpus in the semantic mask is computed through connected components analysis. Cutouts of fixed size (248, 304, and 64), with an up-sampled resolution of (0.75, 0.75, and 1.65) and orientation (posterior, inferior, and right), are created around these centers. For each cutout, this second model predicts the three vertebrae around the cutout’s center. During this process, each vertebra appears in multiple cutouts (Fig. 4).

We compute the average Dice score for each vertebra across these appearances. Subsequently, vertebra instances are integrated into the final instance mask from the highest to lowest average Dice score. This approach ensures the least consistent predictions are addressed last, minimizing the potential impact of erroneous predictions on neighboring instances. Notably, this reduces the likelihood of skipping an entire vertebra or merging two vertebrae instances. Significantly, our method relies solely on the semantic mask as input to the instance model. Therefore, the image data is not utilized during this stage. Finally, IVDs and endplate structures from the semantic mask are added to the instance mask and are given instance labels based on the nearest vertebra instance above.

For the training, we used an Nvidia A40 for one GPU day. The detailed configurations and pre-processing used for training each model are shown in Appendix A (Electronic Supplementary Material). One inference run of SPINEPS with both models on a whole-body scan of NAKO with shapes (501, 914, and 16) takes about 50 s to process. This was tested on a separate machine using Ubuntu 22.04 with a GeForce RTX 3090, an AMD Ryzen 9 5900×, and 128 GB RAM. After both semantic and instance segmentation, post-processing techniques are employed.

### Post-processing

Voxels in the instance mask that are zero in the semantic mask are removed. Furthermore, each connected component present in the semantic mask, but missing in the instance mask, is assigned to the instance with the most neighboring voxels. This ensures consistency in foreground voxels between both masks. A bounding box analysis is used to remove elements isolated from the largest connected component (i.e., the target spine). Additionally, for each articularis inferior and superior connected component, the instances are relabeled based on majority voting. The instance model mostly mixes the neighboring vertebrae instances in those regions by just a few voxels. This ensures consistency and clean edges.

### Experiments

This study used the nnUNet approach from a previous study as the baseline model [22]. We compared its performance to our SPINEPS approach, training solely on the SPIDER dataset and evaluating the SPIDER test split. As the SPIDER ground truth contains only an instance mask, we compare our instance mask output with it. To enable a semantic evaluation, we derive the anatomic group by its instance, i.e., all vertebra instances receive the same label, before calculating the metrics.

Our approach trained only on the automated annotations of the NAKO training data is evaluated on the manually corrected NAKO test set and in-house data to demonstrate the effectiveness of the automated annotations. Additionally, the performance is compared to a model trained on both NAKO training data and the SPIDER dataset. As only the semantic masks were manually corrected for the test set, only the semantic output of the approach was evaluated. We also omitted evaluations for the sacrum and endplate structure, because they were not part of the manual annotation process of the test data.

### Statistical analysis

For evaluation, the Dice similarity coefficient (DSC) and the average symmetric surface distance (ASSD), indicating average distances from segmented edges to reference annotations, is employed. Instance-wise metrics—recognition quality (RQ), segmentation quality (SQ), and panoptic quality (PQ), as described in [29], calculated using panoptica [30]—provided insights into instance prediction performance. Instances with an intersection over a union greater than or equal to 0.5 were considered true positives.

Statistical significance was determined using the Wilcoxon signed-rank test on Dice and RQ metrics, with *p* < 0.05 indicating statistical significance.

## Results

Table 1 presents the demographic and clinical characteristics of the subjects. Out of the total 2030 subjects from the NAKO subset, automated annotations for 1477 (mean age 53, 49% female) were created. We observed no disease-related pattern in our exclusion set, like strong scoliosis or hyper-intense spots. Data from the public SPIDER or the in-house dataset were not excluded (Fig. 1).

### Performance

**Fig. 5:**
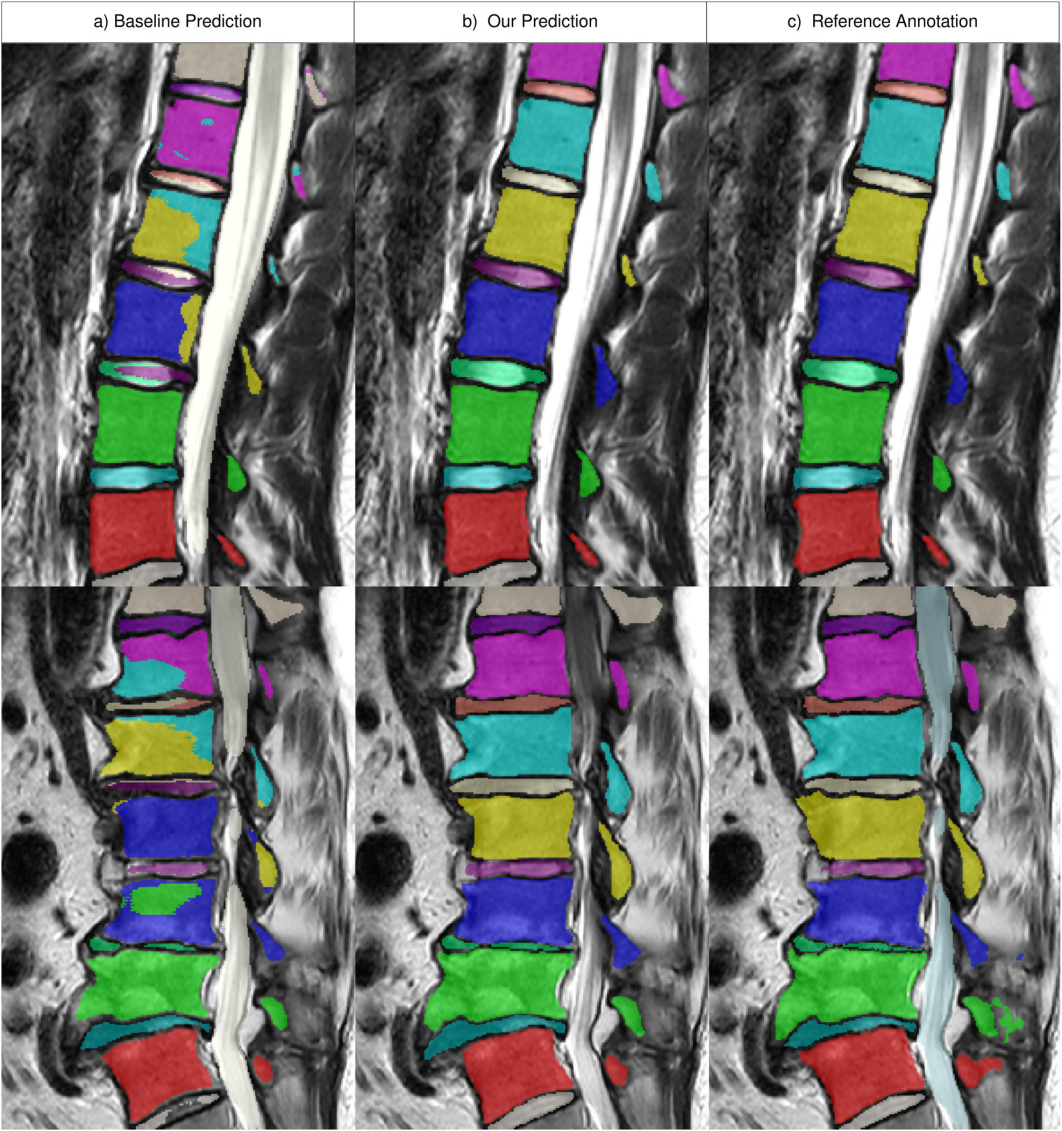
Example from the SPIDER test set. Example subjects where the baseline (**a**) produces a typically found error: mixing different instance labels. Our approach (**b**) is very close to the reference annotation (**c**). This type of error the baseline made did not occur with our approach

SPINEPS outperforms the baseline across all metrics and structures (e.g., vertebra instance ASSD 0.21 vs 0.435; all dice *p* < 0.001). This is true for both the semantic comparison (structure-wise) and the instance metrics (Table 2). Even without the proposed optional post-processing techniques, our two-phase approach outperforms the baseline (e.g., vertebra instance DSC 0.922 vs 0.911, *p* < 0.001). Contrary to the baseline, our model does not produce global instance segmentation errors (Fig. 5). Additionally, the lower standard deviation and the overall lower ASSD values suggest a higher robustness of our technique. Finally, our approach is not only better on average, but consistently across all test samples.

**Table 2:** Performance comparison to baseline

Structure	Metric	nnUNet baseline	SPINEPS w/o post-processing (ours)	SPINEPS (ours)
Global structure-wise				
Vertebra	↑ DSC	0.927 ± 0.026	**0.942** ± **0.022**	See SPINEPS w/o post-processing
IVD	↑ DSC	0.891 ± 0.036	**0.907** ± **0.033**	
Spinal canal	↑ DSC	0.924 ± 0.03	**0.937** ± **0.025**	
Average	↑ DSC	0.91 ± 0.02	**0.929** ± **0.019**	
Instance-wise				
Vertebra	↑ DSC	0.911 ± 0.1	0.922 ± 0.081	**0.933** ± **0.086**
	↑ RQ	0.972 ± 0.041	0.988 ± 0.033	**0.992** ± **0.03**
	↑ SQ	0.85 ± 0.074	0.866 ± 0.041	**0.882** ± **0.03**
	↑ PQ	0.827 ± 0.086	0.855 ± 0.049	**0.882** ± **0.074**
	↓ ASSD	0.435 ± 0.872	0.28 ± 0.303	**0.21** ± **0.175**
IVD	↑ DSC	0.877 ± 0.095	0.901 ± 0.066	See SPINEPS w/o post-processing
	↑ RQ	0.978 ± 0.04	0.983 ± 0.053	
	↑ SQ	0.794 ± 0.083	0.824 ± 0.054	
	↑ PQ	0.777 ± 0.091	0.819 ± 0.056	
	↓ ASSD	0.486 ± 1.08	0.261 ± 0.275	
Average	↑ DSC	0.894 ± 0.024	0.912 ± 0.15	**0.917** ± **0.023**
	↓ ASSD	0.461 ± 0.036	0.271 ± 0.013	**0.236** ± **0.036**

The performance comparison between the nnUNet baseline adapted from a previous study [22] and our SPINEPS approach on the test split of the SPIDER dataset. To fairly compare how much our post-processing systems contribute, the metrics of SPINEPS without post-processing are also shown. We did not employ any post-processing for the semantic model, and it does not influence the IVD instance prediction. Our approach outperforms the baseline in every metric, especially in the instance-wise metrics. The largest difference can be seen in the instance-wise vertebra ASSD metric, where our approach plus one standard deviation is still better than the average of the baseline. Mean and standard deviations are reported. The arrows before the metric name indicate if smaller or higher values are better. The best results in the comparison are marked in bold

*IVD* intervertebral disc, *DSC* Dice similarity coefficient, *RQ* recognition quality, *SQ* segmentation quality, *PQ* panoptic quality, *ASSD* average symmetric surface distance

A model trained on both the NAKO automated annotations as well as the SPIDER dataset yields a DSC of 0.911 for vertebrae, 0.951 for IVDs, and 0.922 for the spinal canal on our test set of 75 randomly chosen, manually corrected subjects (Table 3). Training with both datasets outperformed the model only trained on NAKO (all dice *p* < 0.001). When evaluated on the individual regions (cervical, thoracic, and lumbar), our approach demonstrates its best overall performance in the thoracic region and its weakest in the cervical region, as shown in Appendix B: Performance by Region.

**Table 3:** Performance on NAKO test set

Structure	Metric	SPINEPS (NAKO)	SPINEPS (NAKO + SPIDER)
Global structure-wise			
Vertebra	↑ DSC	0.894 ± 0.034	**0.911** ± **0.034**
IVD	↑ DSC	0.948 ± 0.02	**0.951** ± **0.021**
Spinal canal	↑ DSC	0.904 ± 0.045	**0.922** ± **0.032**
Spinal cord	↑ DSC	0.928 ± 0.072	**0.939** ± **0.051**
Average	↑ DSC	0.918 ± 0.024	**0.931** ± **0.018**
Vertebra substructures			
Arcus vertebra	↑ DSC	0.835 ± 0.063	**0.853** ± **0.061**
	↓ ASSD	0.365 ± 0.227	**0.301** ± **0.186**
Spinosus process	↑ DSC	0.781 ± 0.054	**0.813** ± **0.056**
	↓ ASSD	0.5 ± 0.275	**0.396** ± **0.24**
Articularis inferior	↑DSC	0.747 ± 0.096	**0.763** ± **0.096**
	↓ ASSD	0.54 ± 0.512	**0.497** ± **0.44**
Articularis superior	↑ DSC	0.735 ± 0.103	**0.741** ± **0.103**
	↓ ASSD	0.569 ± 0.51	**0.545** ± **0.431**
Costal process	↑ DSC	0.639 ± 0.109	**0.698** ± **0.108**
	↓ ASSD	1.39 ± 1.07	**1.03** ± **0.898**
Vertebra corpus	↑ DSC	0.934 ± 0.021	**0.948** ± **0.023**
	↓ ASSD	0.398 ± 0.18	**0.287** ± **0.36**
Average	↑ DSC	0.778 ± 0.1	**0.803** ± **0.09**
	↓ ASSD	0.626 ± 0.381	**0.51** ± **0.277**

The performance comparison between SPINEPS trained only on the NAKO dataset and SPINEPS trained with both the NAKO and the SPIDER dataset. Evaluation is done on the manually corrected test split from the NAKO and in-house dataset. Incorporating the manually annotated SPIDER dataset improves each metric. Mean and standard deviations are reported. The arrows before the metric name indicate if smaller or higher values are better. The best results in the comparison are marked in bold

*IVD* intervertebral disc, *DSC* Dice similarity coefficient, *ASSD* average symmetric surface distance, *NAKO* German National Cohort

**Fig. 6:**
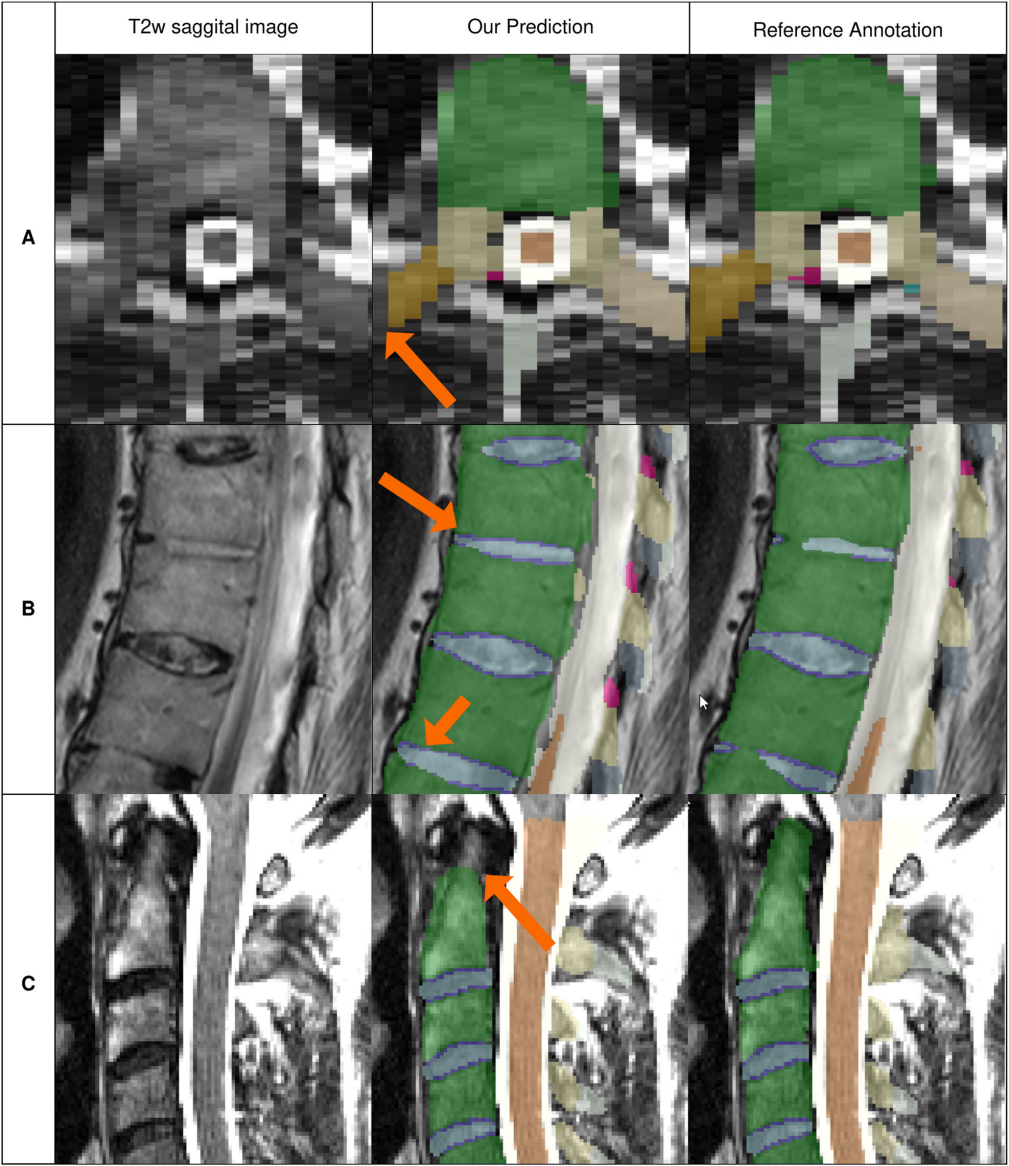
Typical SPINEPS errors. Showcase of the most common mistakes made by our approach trained on both NAKO and SPIDER. Arrows indicate the area of erroneous prediction. **a** An axial snapshot that shows that our approach tends to miss the outermost voxels in the left/right direction, especially for the coastal process structure (orange). In very aberrant cases with partially merged vertebrae (**b**), our approach tends to over-segment the IVDs. **c** The dens axis structure of the C2 is not fully segmented. We hypothesize these issues mostly come from the imperfect translation of MR to CT and subsequent loss of CT segmentation quality

We also assessed the most common errors the models trained on NAKO and SPIDER made (Fig. 6). The model struggles to properly annotate the outermost slices in the left/right dimension. This leads to pixel omissions in the processus costalis/transversus structures. In highly aberrant cases, especially in merged vertebrae, the transition between IVD and corpus often has local errors. Lastly, our models struggle to fully segment the dens axis structure of the C2.

## Discussion

This work presents the first publicly available model for whole spine segmentation in sagittal T2w MR images. Training a model on a combination of automatically generated annotations generalized with good performance. Our approach yields both a semantic and an instance mask. Additionally, this study demonstrated that our two-phase approach yields improved semantic and instance segmentation capabilities compared to a nnUNet trained on instance references directly, as described in a previous study [22].

Spinal segmentation has been addressed by a multitude of approaches. The TotalSegmentator from Wasserthal et al [31] enables the segmentation of more than a hundred classes. However, it works only in CT, and according to their assessment, the most common errors it produces are the confusion of instance labels. They used a nnUNet trained directly on instance labels similar to our baseline [22, 26]. This study presents samples where the baseline produces segmentation errors by confusing different instance labels. We hypothesized that the different labels have the same semantic structure and look similar to the model. Our approach avoids this by first segmenting semantically and using the proposed sliding window technique on fixed cutout sizes. This simplifies the instance segmentation task for the model and limits possible prediction errors to a local influence. The significant increase in metrics evaluating instance segmentation confirms this.

The commercially available CoLumbo software [32] segments the vertebral body, spinous and disc structures among other in the lumbar region. By dividing each vertebra into substructure labels, our approach enables a more detailed automatic analysis of the whole spine. Most other approaches either work on a specific region, like the SPIDER baseline [22], and fail to capture arbitrary fields of views [12] or don’t include the posterior elements [14]. Using the translation approach, this study successfully transferred vertebra substructure segmentations into MRI without manually annotating a single image.

Our approach is trained on a large cohort of MRI data from the NAKO [23]. Such a segmentation technique enables further studies and essential analysis, such as deriving normative values, as shown by Streckenbach et al [25]. However, in that study, the proposed model only segments the vertebra corpus, IVD, and spinal canal region semantically. Unlike our method, further algorithms are required to derive the instance masks from their semantic outputs, rendering downstream tasks more challenging.

Nevertheless, our study has limitations. The population of the NAKO is derived from an average, healthy German population. Thus, compared to a typical hospital dataset, pathologies may be under-represented, and despite including pathological cases in our test data, the same performance cannot be guaranteed on out-of-distribution imaging data, such as post-operative MRI.

Our instance segmentation does not incorporate a labeling step, meaning an instance label in SPINEPS output does not correspond to the same vertebral body across subjects. Instead, the instance labels are counted from top to bottom regardless of the field of view or the presence of enumeration abnormalities, such as 13 thoracic vertebrae [33]. Future research could entail a labeling step after our two-phase segmentation approach to label the vertebra instances anatomically correct, as it has been similarly done in CT [34].

The T2w sagittal images from the NAKO often contain only a few slices and have a slice thickness of 3.3 mm. Our model occasionally encounters difficulty accurately segmenting the outermost voxels along the left/right dimension. This leads to pixel omissions in the processus costalis/transverse structures, making it the least proficient substructure in our model’s performance. In a preliminary test of training only on the manually corrected test data, a reduction in these issues was observed. This suggests that these errors arose from the training data distribution. Therefore, to reduce those errors in the future, we could fine-tune our model on manually corrected data, preferably on wider MR images.

In conclusion, this study presented SPINEPS, a two-phase semantic and instance segmentation approach, which is superior to an nnUNet baseline trained for instance segmentation. We demonstrated our approach can generalize well using automatic annotations only, partially derived from an MR-to-CT translation approach. Finally, a whole spine model was presented to accurately segment 14 spinal structures in T2w sagittal scans in cervical, thoracic, and lumbar regions, both semantically and instance-wise. The models and approaches are made publicly available^1^.

## Supplementary information

ELECTRONIC SUPPLEMENTARY MATERIAL

## Abbreviations

ASSD: Average symmetrical surface distance
DSC: Dice similarity coefficient
IVD: Intervertebral disc
NAKO: German National Cohort
PQ: Panoptic quality
RQ: Recognition quality
SQ: Segmentation quality
SPINEPS: Spinal phase-wise imaging network for paired segmentation
TSE: Turbo spin echo

## Footnotes

1 https://github.com/Hendrik-code/spineps.
