# Supplementary material for: SPINEPS—automatic whole spine segmentation of T2-weighted MR images using a two-phase approach to multi-class semantic and instance segmentation
Source: Eur Radiol. 2024 Oct 29;35(3):1178–89. doi: 10.1007/s00330-024-11155-y (PMC11836161; doi:10.1007/s00330-024-11155-y)
Supplement: Supplementary file 1 — ELECTRONIC SUPPLEMENTARY MATERIAL [file 330_2024_11155_MOESM1_ESM.pdf]

SPINEPS – Automatic Whole Spine Segmentation of T2-weighted MR images using a Two-Phase Approach to Multi-class Semantic and Instance Segmentation.

ELECTRONIC SUPPLEMENTARY MATERIAL

Appendix A: Training Procedure

The data was prepared by reorienting each image consistently and re-sampling them to the same resolution. For the experiments using only SPIDER, we used the resolution suggested by the nnUNet framework (27). For the NAKO-based model, we used an upsampled resolution of (0.75, 0.75, 1.65), where the third dimension is the left/right axis. When training with the NAKO images, we cropped them slightly in each dimension. This reduced the errors made by the automatic annotations process. Additionally, the T2w images were pre-processed by applying the N4 Bias field correction algorithm (36).

As the annotations in the SPIDER dataset (23) only consist of instance labels, an in-house available segmentation model based on (29) was used to create three subregion labels for the vertebrae. This was only utilized for training purposes.

This study utilized the widely used nnUNet (27) as semantic model on the combined annotations. As the annotations are not rotation-invariant, we disabled the Mirroring Augmentation. Besides setting the patch size to (256, 256, 64), the automatically calculated parameters from the nnUNet framework were used. We trained for 1000 epochs, a batch size of 2, and with 3-fold cross-validation on the 3D fullres setup on a Nvidia A40. Each fold roughly required one GPU day. The baseline was trained identically, but on the instance-labels, not the semantic ones. For inference, all 3

folds were run and then their outputs averaged. No other ensemble techniques were incorporated.

For the instance phase, a cutout size of (248, 304, 64) with orientation (posterior, inferior, right) was chosen to always contain at least three complete vertebrae in the same target upsampled resolution of (0.75, 0.75, 1.65). We trained a UNet3D model on these cutouts to segment the three vertebrae around the center with the labels 1 (above), 2 (center), and 3 (below). We used a batch size of 2, a learning rate of 1e-4 that decreases linearly each epoch to 1e-6, and trained for 300 epochs. For augmentations, we used random scaling in the range [-0.2,0.2], Random Erosion, Random Down- and then Upsampling, and Random Labeldrop, each with a 10% chance of occurring during training. With a 25% chance, we adopted Random Vertical Crop. Additionally, Horizontal Flip was used as augmentation, practically doubling the training data. When flipping, we made sure the left/right related labels are also flipped.

Appendix B: Performance by Region

We compared the performance of our best model for each region individually (Table S1). Each German National Cohort (NAKO) subject has scans for the cervical, thoracic, and lumbar spine. Our in-house dataset does not. From the 75 subjects, we have 72 images in the cervical and thoracic regions, and 75 in the lumbar region. Our trained model overall performs best on the thoracic region, followed by the lumbar and then the cervical region. We hypothesize the cervical regions to be worse because the substructures of the vertebrae are very difficult to distinguish in T2w sagittal TSE images there. Even the CT segmentations that were used were performing worse in the upper cervical region, further hinting at this underlying problem.

Table S1. Performance on German National Cohort Test set by region

Structure	Metric	Cervical region	Thoracic region	Lumbar region
		**Global structure-wise**		
Vertebra	↑ DSC	0.875 ± 0.026	0.925 ± 0.014	**0.931 ± 0.026**
IVD	↑ DSC	0.942 ± 0.023	**0.958 ± 0.017**	0.952 ± 0.019
Spinal Canal	↑ DSC	0.920 ± 0.037	**0.938 ± 0.030**	0.918 ± 0.027
Spinal Cord	↑ DSC	**0.955 ± 0.025**	0.952 ± 0.032	0.913 ± 0.07
		**Vertebra substructures**		
Arcus Vertebra	↑ DSC	0.812 ± 0.047	**0.885 ± 0.03**	0.863 ± 0.07
	↓ ASSD	0.415 ± 0.186	**0.21 ± 0.073**	0.28 ± 0.20
Spinous process	↑ DSC	0.773 ± 0.043	0.828 ± 0.035	**0.836 ± 0.062**
	↓ ASSD	0.52 ± 0.252	**0.326 ± 0.145**	0.34 ± 0.258
Articularis inferior	↑ DSC	0.676 ± 0.09	0.803 ± 0.042	**0.81 ± 0.077**
	↓ ASSD	0.805 ± 0.589	**0.282 ± 0.102**	0.404 ± 0.268
Articularis superior	↑ DSC	0.65 ± 0.087	**0.801 ± 0.056**	0.772 ± 0.091
	↓ ASSD	0.817 ± 0.449	**0.285 ± 0.13**	0.531 ± 0.44
Costal process	↑ DSC	0.64 ± 0.1	**0.750 ± 0.093**	0.704 ± 0.099
	↓ ASSD	1.51 ± 0.93	**0.734 ± 0.54**	0.86 ± 0.95
Vertebra corpus	↑ DSC	0.932 ± 0.025	**0.953 ± 0.014**	**0.959 ± 0.019**
	↓ ASSD	0.267 ± 0.16	**0.266 ± 0.099**	0.324 ± 0.583
